# In Vitro Study on the Influence of the Buccal Surface Convexity of the Tooth upon Enamel Loss after Bracket Removal

**DOI:** 10.3390/ma17071519

**Published:** 2024-03-27

**Authors:** Sandra Pallarés-Serrano, Alba Pallarés-Serrano, Antonio Pallarés-Serrano, Antonio Pallarés-Sabater

**Affiliations:** 1 Doctoral School, Catholic University of Valencia, 46001 Valencia, Spain; antonio.pallares@ucv.es; 2 Department of Orthodontics, School of Medicine and Dentistry, Catholic University of Valencia, Quevedo 2, 46001 Valencia, Spain; alba.pallares@ucv.es (A.P.-S.); antonio.pserrano@ucv.es (A.P.-S.)

**Keywords:** orthodontics, dental convexity, dental debonding, dental polishing

## Abstract

Polishing after the removal of brackets is the final step in orthodontic treatment. It is simple to perform, though some studies have reported that polishing causes damage to the enamel surface. An in vitro study was made of the influence of the buccal surface convexity of the tooth upon possible enamel loss when the remaining resin and adhesive are removed after bracket decementing using two different polishing modes: a tungsten carbide bur at low and high speeds. The convexity of the buccal surface was quantified in 30 incisors and 30 premolars. A stereoscopic microscope was used to obtain photographs of the profile of the crown, and Image J software was used to calculate convexity by dividing the length of a line from the cementoenamel junction to the incisal margin by another line from the mentioned junction to the maximum convexity of the buccal surface. Brackets were cemented on all the teeth and were decemented 24 h later. In both groups, the residual composite was removed with a tungsten carbide bur at a low speed in one-half of the teeth and at a high speed in the other half. The buccal surface of each tooth was then photographed again, and the convexity was calculated and compared against the baseline value. The difference between the two values were taken to represent the enamel loss. The convexity of the premolars was significantly greater than that of the incisors, but this did not result in greater enamel loss when the same polishing mode was used. However, the tungsten carbide bur at a high speed proved more aggressive, causing significantly greater enamel loss than when used at a low speed.

## 1. Introduction

Orthodontic treatment is completed by the decementing or debonding of the brackets, buttons and other elements adhered to the enamel surface, with subsequent elimination of the remaining adhesive and composite on the enamel surface [1]. This latter step is very important, since failure to eliminate all such material traces on the enamel may create retention zones that can accumulate bacteria, leading to demineralization and carious lesions, as well as produce enamel damage with permanent sequelae affecting both the aesthetics and the integrity of the tooth [2]. In this regard, since enamel is a tissue that lacks a regenerative capacity, its preservation is of special concern [3,4,5].

For decades, tooth damage has been investigated using indices that assess the enamel roughness caused by the use of burs (surface roughness index [SRI]) [6] and the efficacy of burs in removing adhesive (adhesive remnant index [ARI]) [7] and composite (composite remnant index [CRI] [6]). Although it seems unavoidable for the enamel to be affected by adhesive removal, the damage may be reduced provided adequate materials are used. In this regard, the prevention of enamel loss is complicated by the great variety of techniques that are currently available: tungsten carbide burs, diamond burs, Arkansas burs, fiberglass burs and composite burs, among others, as well as high- and low-speed rotary instruments [8,9,10,11,12].

Although most studies in this field are based on qualitative indices [6,7,8], in recent years quantitative studies have also been carried out to an attempt to ascertain the volumetric loss of enamel [13] in order to produce evidence as to which may be the best technique, although there is considerable controversy in the literature and no standardized protocol has been established to date [14,15,16]

Few studies have addressed this issue, and the published results are very heterogeneous, with the use of different methodological approaches that make it difficult to establish comparisons [9,14,17,18,19,20,21].

Likewise, the great majority of the published studies do not take into account the convexity of the buccal surface of the incisors or premolars to which the brackets are adhered, despite the potential impact of the anatomical differences of the buccal surfaces between the two types of teeth [6,11,22,23].

The present in vitro study evaluates the influence of the buccal surface convexity of the tooth upon enamel loss after bracket removal, considering the operating speed of the tungsten carbide bur. The following null hypotheses were established: 1. The initial (baseline) convexity of the buccal surface does not influence the enamel loss. 2. The operating speed of the tungsten carbide bur in removing the composite material does not influence the enamel loss. 

## 2. Materials and Methods

### 2.1. Study Design

An in vitro, randomized and controlled study was carried out, supervised and authorized for the use of organic tissues by the Ethics Committee of the Catholic University of Valencia (Valencia, Spain) (protocol code: UCV/2020-2021/170). All patients gave informed consent to the use of the teeth for the present study. 

### 2.2. Sample Size

In calculating the sample size, a significance level of 5% was considered, with a statistical power of 80% and a standard deviation (SD) of 2.5%. The minimum sample size was 25 teeth per comparator group. The estimated percentage of losses was 15% (broken or deteriorated teeth, teeth detached from the working support, etc.). The final sample size per group was thus taken to be 29 teeth, and although this number was further optimized to 35, it was finally rounded to 30 teeth to ensure a balance between the groups after taking into account teeth broken at bracket removal or during the polishing process. The software GRANMO version 7.12 was used to assess the sample size of specimens per group number.

### 2.3. Sample Preparation

**Figure 1:**
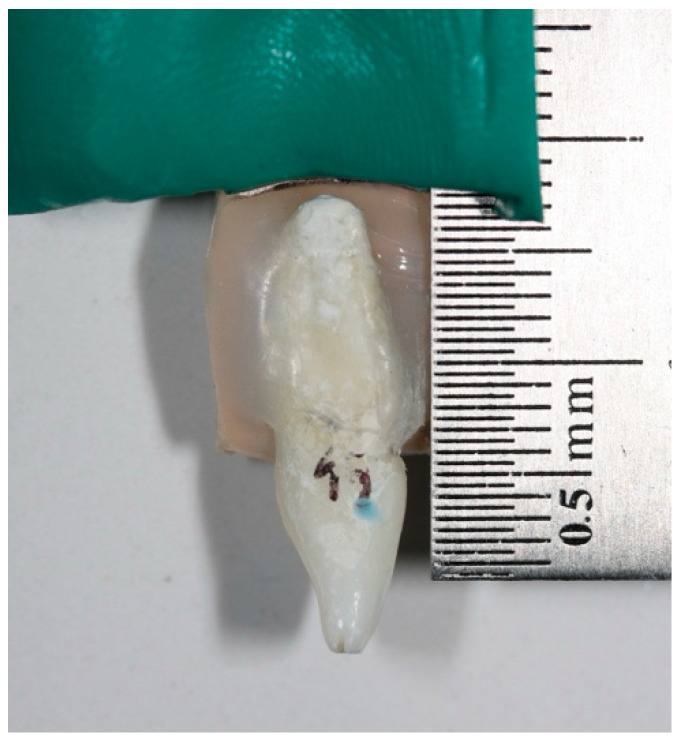
Tooth on the flat support inserted in the silicone block.

Two groups of teeth with different convexities were established: 30 incisors and 30 premolars. All the teeth were intact and had been removed for orthodontic or periodontal reasons. The specimens were cleaned, removing dental calculus, and were kept in saline solution at room temperature until use. Convexity was objectively assessed based on profile photographs of the teeth. The 60 teeth were previously fixed with transparent silicone, adhering the root of the tooth onto a flat support measuring 2 × 8 × 1 cm in size. The support in turn was inserted in a silicone block (Figure 1). 

**Figure 2:**
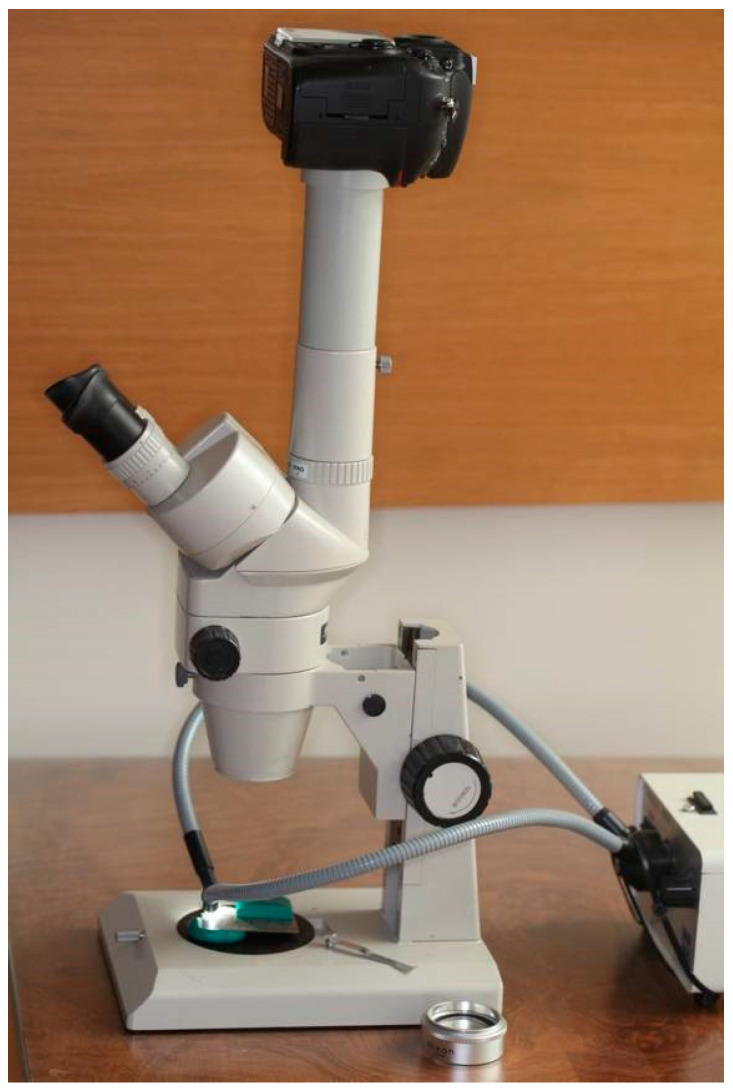
Stereoscopic microscope (Nikon SMZ 2T) and camera (Nikon D800).

The clinical crown was left exposed, in order to be able to reproduce the same positions at the time of obtaining the photographs with a stereoscopic microscope Nikon SMZ- 2T (Nikon, Tokyo, Japan) and camera Nikon D800 (Nikon, Tokyo, Japan) (Figure 2).

**Figure 3:**
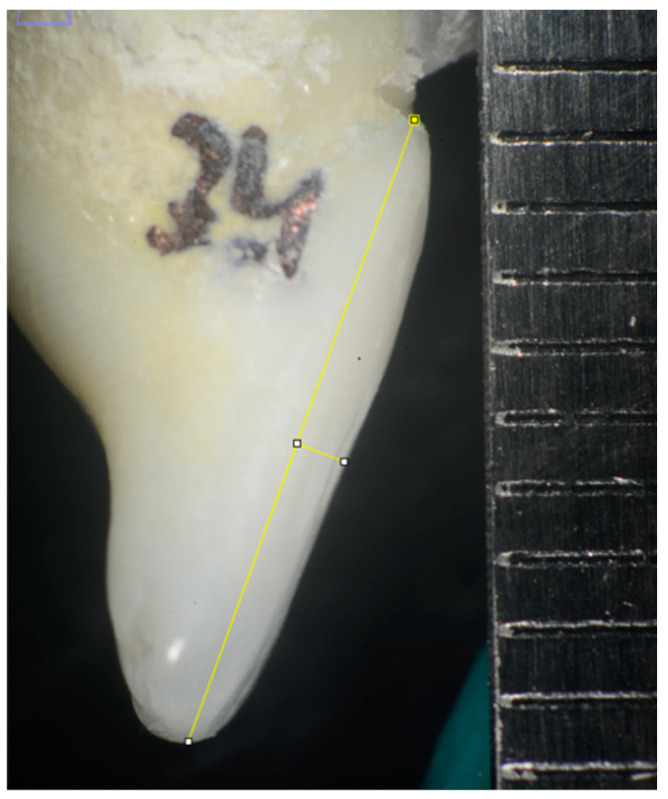
The ratio between the lengths of line A and line B provides the convexity of the incisors to obtain a convexity index.

**Figure 4:**
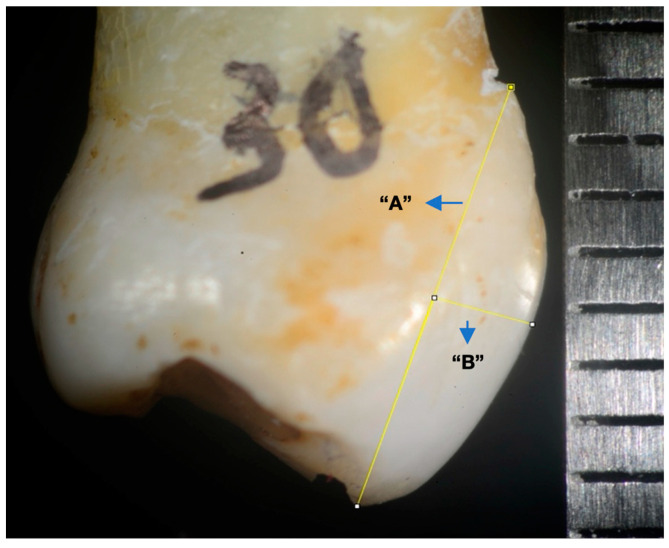
The ratio between the lengths of line A and line B provides the convexity of the premolars to obtain a convexity index.

In order to perform the measurements, the photographs included a millimeter ruler for scaling with the Image J application (version 1.54f). Image J was created by Wayne Rasband, and it is an open-source Java-based image processing program developed and designed by the national institutes of health (NIH) for the analysis of scientific images [24,25]. On the images obtained, we marked a line from the cementoenamel junction (CEJ) to the incisal margin (line A) and another line from the mentioned junction to the maximum convexity of the buccal surface (line B) (Figure 2). The surface convexity was obtained as the ratio between the lengths of line A and line B. Accordingly, the smaller the ratio, the greater the convexity and vice versa (Figure 3 and Figure 4).

### 2.4. Procedure

All teeth were subjected to acid etching with 37% orthophosphoric acid (Dentsply, York, PA, USA) during 30 s on the buccal surface at the center of the clinical crown, in accordance with the size of the base of the metal bracket (Dentsply, Sirona OmniArch^®^, Court East, Sarasota, FL, USA). After etching, the teeth were washed with water for 15 s and air-dried until a chalk-white color was observed. Adhesive (Transbond XT Adhesive Primer, 3M ESPE, St. Paul, MN, USA) was then applied to the acid-etched enamel, and the composite resin (Transbond XT Adhesive Paste, 3M ESPE, St. Paul, MN, USA) was applied to the base of the bracket.

Bracket holding forceps were used to press the bracket into the buccal surface of the tooth at the center of the clinical crown, and a probe was used to remove the excess composite around the bracket. Photopolymerization was applied for 5 s on each side of the bracket using a wireless LED photocuring lamp (Ivoclar Vivadent, Bluephase^®^ Style 20i, Ringwood, Australia) with an irradiancy of approximately 1700 mW/cm^2^.

All the samples were stored in water at room temperature for 24 h, after which the brackets were decemented with bracket removing pliers. For the removal of the residual adhesive and resin, the 30 incisors and 30 premolars were randomized to two groups. In one group, the removal was carried out with a 30-lamina U212 tungsten carbide bur (AXIS Dental Sárl, Crissier, Switzerland) using a high-speed handpiece (Turbine T1 Boost Dentsply Sirona), while in the other group we used a tungsten carbide bur with a low-speed handpiece (X95L Ti Max: Contra Angle Dental 1:5—NSK), in order to compare the same bur at different speeds and assess the impact upon the convexity of the tooth in the form of enamel loss (Table 1). 

A standardized technique was used in which the burs were placed parallel to the long axis of the tooth, performing lateral movements in the mesiodistal direction of the crown until all the remaining adhesive and composite was visually seen to have been eliminated, in a way similar to clinical practice.

In order to standardize the laboratory process and maximize the bur operating efficiency, the burs were replaced every 10 teeth in order to prevent blunting effects caused by contact with hard tissues, such as healthy enamel and the composite material used in orthodontics [23].

### 2.5. Evaluations and Measurements

After polishing, each tooth was returned to the silicone block and photographed again, with its convexity calculated using the Image J application. The values obtained were entered in an MS Excel spreadsheet, with the calculation of the difference between the two convexities as a measure of enamel loss.

### 2.6. Statistical Analysis

Since the sample size was large enough (*n* ≥ 30), we used the Student *t*-test for the comparison of independent samples. The statistical analysis was performed with the SPSS version 23 statistical package, using a 95% confidence level and accepting statistical significance for *p* < 0.05 in all cases.

## 3. Results

**Figure 5:**
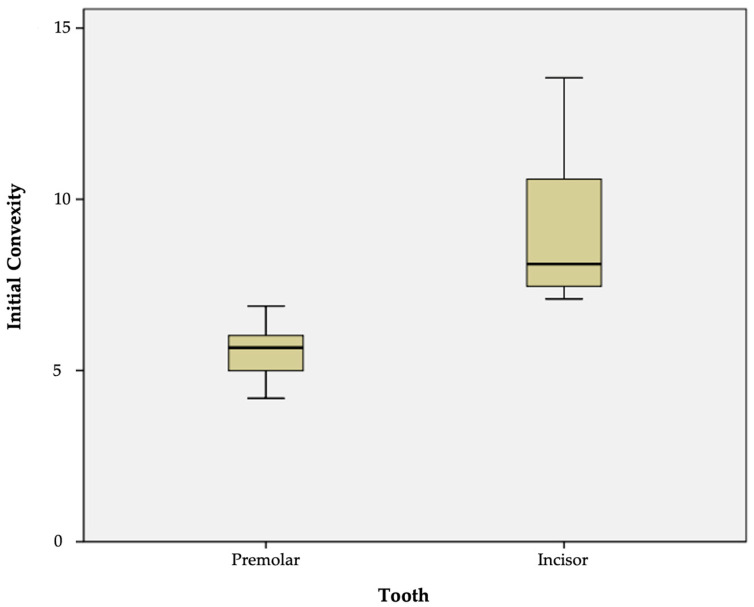
Comparison of initial (baseline) convexity according to the type of tooth.

In relation to the anatomical type of tooth, the mean baseline convexity of the premolars was 5.57 ± 1.87 (range 4.18–6.88) versus 8.98 ± 5.07 (range 7.09–13.55) in the case of the incisors (Figure 5).

In all cases, the convexity ratio corresponding to the premolars was significantly smaller than that of the incisors (*p* < 0.001). Thus, the surface convexity of the premolars was significantly greater than that of the incisors (Table 2).

After polishing with the tungsten carbide bur at a low speed, the mean variation in convexity value versus the baseline was 0.050 ± 0.015 in the case of the premolars and 0.048 ± 0.018 in the case of the incisors (Table 3).

The Student *t*-test showed that the removal of the remaining adhesive and resin with the tungsten carbide bur at a low speed resulted in no statistically significant differences in the variation in surface convexity between the premolars and incisors (*p* = 0.08).

After polishing with the tungsten carbide bur at a high speed, the mean variation in the convexity value versus the baseline was 0.27 ± 0.35 in the case of the premolars and 0.29 ± 0.04 in the case of the incisors. In this case, the Student *t*-test likewise showed no statistically significant differences in the variation in surface convexity between the premolars and incisors (*p* = 0.53) (Table 4).

**Figure 6:**
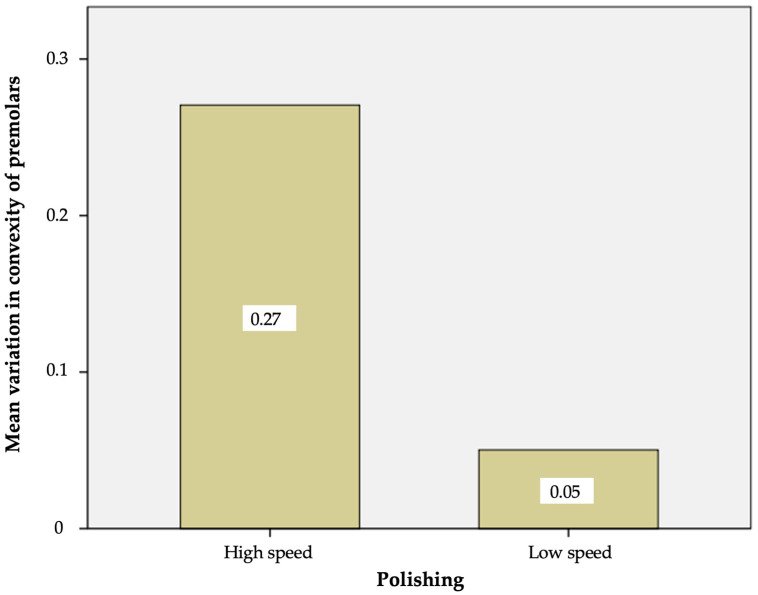
Mean variation in convexity of the premolars with the high- and low-speed tungsten carbide bur.

**Figure 7:**
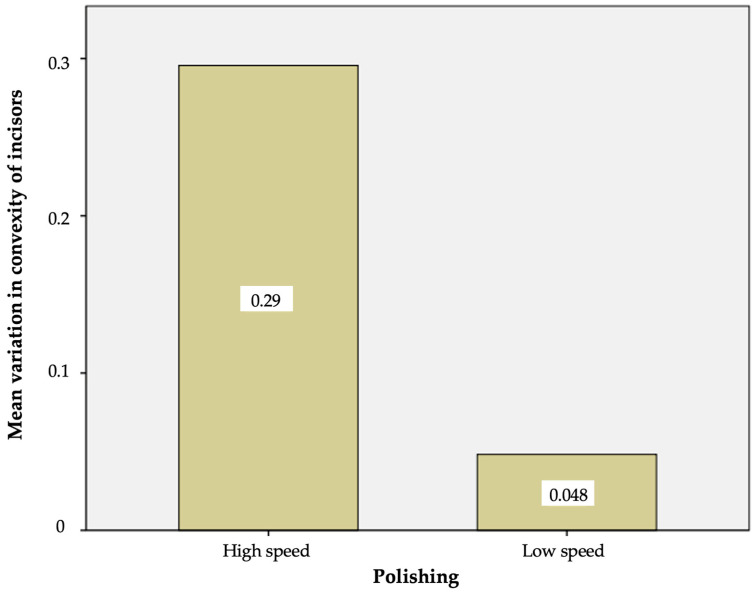
Mean variation in convexity of the incisors with the high- and low-speed tungsten carbide bur.

However, although no significant differences in enamel loss were observed between the two types of teeth when the same polishing mode (low or high speed) was used, the removal of the remaining adhesive and resin with the tungsten carbide bur at a high speed resulted in a significantly greater enamel loss in both the incisors and premolars than when using the tungsten carbide bur at a low speed (Figure 6 and Figure 7).

Thus, the anatomical type of tooth did not exert a statistically significant influence upon the surface convexity after polishing, regardless of the polishing mode involved (low or high speed). However, significant differences were observed for both types of teeth in terms of the influence of polishing upon the variation in surface convexity, with significantly greater variation in both the premolars and incisors when the tungsten carbide bur was used at a high speed. 

## 4. Discussion

The present study was carried out to measure enamel alteration in terms of the variation in convexity of the buccal profile of the crown, recording a numerical value corresponding to the difference in convexity of the tooth between the baseline prior to polishing and the convexity observed after polishing, which can be taken to reflect the thinning of the enamel layer. The initial or baseline convexity of the tooth was regarded as a study variable, since it influences the incidence of the bur on the buccal surface of the tooth. 

Many studies have assessed enamel loss related to the removal of adhesive and composite following the decementing of brackets. A number of different indices have been introduced to measure the damage caused by the bur and to evaluate the best material options in orthodontic practice: composite remnant index (CRI), surface roughness index (SRI) [6], adhesive remnant index (ARI) [7], enamel damage index (EDI) [15] and enamel surface index (ESI) [4], among others [26,27,28,29]. All of these instruments offer assessments based on categorical scales that evaluate visible scratch marks, grooves, abrasions and other irregularities on the enamel surface, in an attempt to classify enamel destruction. In this regard, both light microscopic and scanning electronic microscopic (SEM) techniques have been used [27,28,29,30].

Some authors have used profilometry to measure the depth of the grooves [17,27,28], while others have compared digital models using three-dimensional scanners to observe changes in the anatomy or measure the depth of cracks and grooves [30,31,32]. In turn, other studies have made use of atomic force microscopy (AFM) [22,33,34]. 

The studies found in the literature do not take into account the convexity of the buccal surface where the brackets are adhered, which is exposed to the action of the bur during removal of the remnant adhesive and composite material. The existing articles generally focus on comparing and classifying the aggressivity of bur action upon the enamel layer but do not analyze the possibility that teeth with different anatomical features may be affected differently by the use of the bur. To date, no articles have explored the way in which different materials can affect the convexity of teeth.

Schuler and Van Waes [15] analyzed full arches and found that the more distal the position of a given tooth, the more severe the apparent damage caused by the bur in removing the remnant resin and composite following bracket removal—though the results of the study offered no concrete data in this regard.

Gwinnett and Gorelik [35] in turn reported that the problem is greater in the case of flat anterior teeth than in more curved posterior teeth, but here again the results of the study offered no concrete data. In the present study, we found that buccal convexity was not associated with significant differences in enamel loss between incisors and premolars, at least with the polishing systems used.

The authors also analyzed the Influence of the operating speed (high or low) of the tungsten carbide bur. The methodological design employed afforded objectiveness, since the recording of numerical differences between baseline and post-bracket removal avoided having to depend on the subjective interpretation of images. However, we likewise found no previous studies in the literature using the Image J application or the convexity index, which we developed for use in our study.

Our results evidenced the existence of statistically significant differences (*p* < 0.05) according to the speed of the tungsten carbide bur, with high operating speeds causing a greater loss of surface convexity than low speeds, independently of the original convexity profile of the tooth (analogous in incisors and premolars). 

As a limitation of our study, the lack of similar publications implies that comparisons cannot be made. We thus focus our findings on the effects of the two polishing modes upon the enamel surface.

Our results coincide with those of Ahrari et al. [27], who employed profilometry to measure the irregularities caused by different rotary instruments, including the tungsten carbide bur at high and low speeds. These authors found a low speed to cause less damage to the enamel surface, since the resulting surface irregularities were not significant, in contrast to when high speeds were used.

Our data also coincide with those published by Eliades et al., who found the tungsten carbide bur to be the most effective and conservative option for removing the adhesive material [17]. Likewise, Hannah and Smith recorded their most satisfactory results with the tungsten carbide bur operating at a low speed [36]. In turn, Ireland et al. found that the tungsten carbide bur at a low speed resulted in a lesser loss of enamel thickness than when the same instrument was operated at a high speed [37].

Our findings are also consistent with those of Iglesias et al., who reported improved enamel preservation after polishing with the tungsten carbide bur at a low speed [34].

Otherwise, Janiszewska-Olszowska et al., based on an index created to compare the tungsten carbide bur with other methods, found that the tungsten carbide bur had the most destructive effect on enamel loss [12].

Some studies have reported that the fiberglass bur could equal or even improve upon the performance of the tungsten carbide bur at a low speed in terms of minimizing surface enamel damage [10,11,31]. Future studies would be necessary to compare the two types of instruments, the tungsten carbide bur and the fiberglass bur, with a view to establishing a standardized protocol for polishing the enamel surface with minimal damage after the debonding of brackets used in orthodontic practice. It would also be interesting to incorporate the fluorescence-assisted identification technique (FIT) to reduce the amount of enamel loss due to polishing after debonding [38,39].

## 5. Conclusions

The initial premolar buccal convexity was significantly greater than incisor buccal convexity, though the variation in convexity was not dependent upon the anatomical type of tooth but on the polishing mode used. In this regard, the tungsten carbide bur operating at a high speed had a greater impact upon the surface convexity in both premolars and incisors.

## Figures and Tables

**Table 1:** Study groups and materials used.

Group	Bur	Rotatory Instrument
Incisors	U212 (AXIS Dental Sárl, Crissier, Switzerland)	Turbine T1 Boost-Dentsply Sirona Ref.: 372-2035
Premolars		
Incisors	U212 (AXIS Dental Sárl, Crissier, Switzerland)	X95K Ti Max: Contra Angle 1:5-NSK Ref.: C600001
Premolars		

**Table 2:** Student *t*-test for independent samples.

Student *t*-Test							
Confidence Interval 95%							
Baseline Convexity	t	df	*p*-Value	Means Difference	Standard Error	Lower Limit	Upper Limit
Equal variances not assumed	−0.012	0.074	<0.001	−3.41	0.27	−3.95	−2.873

**Table 3:** Descriptive analysis of the mean variation in convexity of the premolars and incisors with the low-speed tungsten carbide bur.

		Descriptive Statistics							
					Confidence Interval 95%				
Low-Speed Tungsten Carbide Bur		N	Mean	Standard Deviation	Standard Error	Lower Limit	Upper Limit	Maximum	Minimum
Convexity variation	Premolar	30	0.05	0.040	0.007	0.03	0.06	0	0.12
	Incisor	30	0.048	0.049	0.009	0.02	0.06	0	0.18

**Table 4:** Descriptive analysis of the mean variation in convexity of the premolars and incisors with the high-speed tungsten carbide bur.

		Descriptive Statistics							
					Confidence Interval 95%				
High-Speed Tungsten Carbide Bur		N	Mean	Standard Deviation	Standard Error	Lower Limit	Upper Limit	Maximum	Minimum
Convexity variation	Premolar	30	0.27	0.14	0.02	0.21	0.32	0.07	0.47
	Incisor	30	0.29	0.16	0.02	0.23	0.35	0.06	0.64

## Data Availability

Data are contained within the article.
